# Ten Simple Rules for Internship in a Pharmaceutical Company

**DOI:** 10.1371/journal.pcbi.1003600

**Published:** 2014-05-08

**Authors:** Bin Chen

**Affiliations:** Division of Systems Medicine, Department of Pediatrics, Stanford University School of Medicine, Stanford, California, United States of America

Students have to choose between industry and academia at the end of their formal education. One great opportunity to assess their ability in industry is to intern in a company during their academic training [1]. Many pharmaceutical companies often offer a 10- to 12-week summer internship program for undergraduate and graduate students. During this program, interns have an opportunity to work with dedicated mentors from the company on real-world industrial problems related to drug discovery and development as well as to participate in other training activities organized by the internship program office. Depending on the particular project and student performance, interns should be able to accomplish one or more short scientific projects. Some interns may be able to present the results of their work in the form of scientific papers or conference talks or even land a job through the training. This short-term working experience in pharmaceutical companies will help students better understand the pharmaceutical industry, learn the process of drug discovery and development, and build a strong network with experts and fellows in the pharmaceutical field, which can positively contribute to future career development. In addition, it will help students to identify if they really enjoy working in industry and help them in choosing a future career after school.

Thus, it is of extreme importance to make full use of this rare opportunity to explore the pharmaceutical field, to nurture yourself, and to prepare for a career after school. Many students often manage to have one or even more internship trainings during their school years. For those who are planning to do an internship, I would like to tell you about my five internship experiences in three big pharmaceutical companies (namely, Novartis, Pfizer, and Merck) during graduate school at Indiana University, Bloomington. The rules and advice also come from my former intern fellows, mentors, and colleagues from pharmaceutical companies. Besides computational biology and chemistry, many of these rules may be applied to other industrial fields as well.

## Rule 1: Start Preparing for an Internship Early

You need to start looking for internship postings and consulting seniors as early as possible. Submissions of internship applications usually start in December, and an offer is typically issued in March of the next year (the dates vary widely among companies). If you are enrolled in a Master of Science (MS) program and planning to intern in the first summer, you should start preparing right after orientation. You can search postings from job search engines (e.g., indeed.com and Monster.com), professional communities (for example, the International Society for Computational Biology (ISCB) for jobs for computational biology, CCL.NET for jobs for computational chemistry, and LinkedIn groups), or company websites. Some websites provide an email-alert service that sends new job postings automatically. Another great source in which to look for internships is a scientific conference organized by the American Medical Informatics Association (AMIA), Intelligent Systems for Molecular Biology (ISMB), the American Chemical Society (ACS), or Bio-IT World.

Early preparation not only gives you more opportunities to find a suitable position, but also lends you a much longer time to prepare your basic skills. If you start searching before December, you may not see job postings for the next year, but you would see those from the last year, which could guide you in skill preparation. Often the postings list basic requirements and preferred skillsets. Those do not change significantly from year to year. For example, cheminformatics-related programs may expect you to have some experience with particular software and tools (e.g., Pipeline Pilot and Spotfire), while bioinformatics-related programs may expect you to know a bit about public databases (e.g., Gene Expression Omnibus [GEO]) and sequence processing tools and pipelines. These skills can be extended through the courses you are/will be taking or through online programs. You do not need to be an expert, but broader experience is always a plus. Don't be afraid if you don't have extensive experience; these skillsets can be gained quickly, assuming you are not afraid of hard work and willing to learn new things.

## Rule 2: Leverage All Your Sources to Be Selected for an Interview

Interviewers often get a pile of resumes for one position, but only a few of them will be selected for an interview. Except for those resumes coming from prestigious schools or labs, most other resumes, particularly from fresh graduate students, do not have much to distinguish them. In that case, how do you make yours stand out? Other than making your profile perfectly match the job description, you can try networking, which is sometimes very helpful in getting an interview. If the interviewers happen to know you or your references, your chances will definitely increase.

As a new student with no connection to industry, what should you do? The best person to consult is your advisor, who knows you best and has many more connections. Many companies would send the job posting to your advisor and also contact your advisor before they made a decision to hire you. Therefore, do make a good impression on your advisor and tell him/her ahead of time that you would like to apply for an internship. The seniors in your group or school are another valuable resource. Ask them (especially those who have interned before) if they know of any openings anywhere or know anybody who could forward your resume to your dream company. In addition, many conferences (e.g., ISMB) have student sessions that allow students and industrial fellows to mingle.

Another possible way to get an internship would be to contact company scientists directly if you know their work and it is in an area of your interest or expertise. They may be able to work through the internship office if they see a fit. They may even have the opportunity to influence the job posting.

## Rule 3: Survey the Interviewer(s) before and during Interview

An interview can be conducted any time after you submit your application. You may have two interviews for a program and often may have one week or more for preparing an interview. The manager or mentor usually conducts the first interview by phone, followed by another phone interview from human resources (HR) if the manager or mentor approves. Therefore, impressing the manager or mentor is key. In addition to being familiar with all the job requirements and each point in your curriculum vitae (CV), knowing your interviewer's background in advance can be very helpful. You may be able to get a glimpse from LinkedIn or from reading the interviewer's publications.

The phone interview is a great chance to learn more about the position and show your interest, curiosity, and excitement about the internship opportunity. Don't be afraid to discuss the projects you will be working on, the sources you may use, or any questions that will demonstrate your interest. If you are able to raise a couple of interesting questions on their recent work, they will definitely be very impressed.

## Rule 4: Specify Your Target at the Beginning of the Program

Congratulations if you get an offer, but keep in mind that three months is a really short time, especially if you want to accomplish an impressive project, so be sure to prepare in advance before you start. You should take one project first and focus on it. Your mentor may already have a detailed proposal or may only have a general idea. Whatever the situation is, at the beginning you have to sit down with your mentor to discuss your expectations of each other and your objectives and then come out with a practical way to achieve them. If you are not able to come out with a concrete plan immediately, literature review is a helpful way to start. Sometimes, you may want to propose an alternative plan if you can foresee some potential problems. It is recommended that you start with a project that may be publishable, as a publication definitely will benefit both you and your mentor.

## Rule 5: Keep to the Timeline

A 12-week program can be divided into three phases: 2 weeks, 6 weeks, and 4 weeks. The first 2 weeks would be taken up by the orientation, group introduction, training, and project planning. In the 9th or 10th week, you would start wrapping up your work, and in the following week, a poster session would be kicked off, so you basically only have about 6 weeks to devote to your work. Thus, you have to make a clear timeline at the beginning and note every day what you will do and what you have done so that your work is kept on track. It is suggested that you have a weekly meeting with your mentor and coworkers to discuss your progress and address any problems right away. Some companies may have a formal midterm evaluation, which can give you valuable feedback on your progress.

## Rule 6: Don't Hesitate to Ask Questions

Industry greatly values teamwork. Other than your mentor, there may be other colleagues involved in your project, so be ready to communicate with them. Since you basically only have 6 weeks or so to devote to your project, don't waste your time working on something that has been done already. Particularly, at the beginning you need many sources to jumpstart your project. Don't be afraid that you will look stupid by asking basic questions. Your goal is to understand the field and project better, work with your teammates to move forward more productively, and enjoy the progress of learning in the meantime. Your mentors should already have reserved some time to answer your questions, but if their schedule is very tight, you may either email your questions or ask for more meetings. They are always able to squeeze some time in for you.

## Rule 7: Have the Confidence to “Sell” Your Project

In big companies, collaboration between diverse groups happens quite often. Many companies even hold regular social events so that scientists from different backgrounds can mingle. Take advantage of these opportunities to talk about your work with others. As many of them don't know your work, being confident will help convince your audience to listen to your work and appreciate it. Some work—for example, developing a tool for analyzing microarray data—may not seem particularly novel to computational biologists, but for many bench biologists, this tool could save time significantly. Timely feedback may not only inspire your work, but may also help establish potential collaboration. Finally, a poster session offered at the end of the internship can be a good place to “sell” your project. Be sure to write down the names of the colleagues who are extremely interested in your work in case your mentor would like to follow-up.

## Rule 8: Expand Your Horizons beyond Your Project

Journal club, seminars, team meetings, intern training sessions, and many other activities organized by the education office provide opportunities to learn about the company and the process of drug discovery and development. Many companies organize a session in the middle of the summer to introduce the company and hold a social event to help interns communicate with company leaders and management teams. The group or department also has regular meetings that allow you to learn about the work of your colleagues. In addition, an internal e-source is extremely valuable in nurturing yourself. Many companies have their own internal wiki or SharePoint sites, from which you can learn about group projects without bothering your colleagues. You can even participate in drug discovery training for free through their e-learning sources. These are not required during an internship but definitely are benefits you should not miss.

## Rule 9: Be Social, Open-Minded, and Curious

Other than communicating with your mentor and coworkers, you should also be active in engaging with other colleagues, either at the lunch table or during casual talk. It is encouraged to make an appointment with other colleagues individually. Just simply say hi to them and inquire whether they have some time to have a chat. Despite the busy schedules of your colleagues, they often would be very happy to find some time for you in order to learn more about you, discuss their projects, and share their working experience. If you are living with other intern fellows, don't miss that social time either. Otherwise, try to find someone, talk with him or her, and share your experience. Be sure to acknowledge all the colleagues who help you either in your poster or in your lab presentation.

## Rule 10: Finishing the Program Does Not Mean the Ending

You will find that time is flying fast, and soon you have to wrap your work up. Due to the short-term working period, it may happen that you could not finish the project as planned, but you need to document your work in an accessible manner so that your colleagues can continue the work without much effort. Be sure to store the data, analysis, documents, and code as specified several days before leaving so that your colleagues have enough time to look at your work and ask you questions face-to-face. Some groups have a source code version control tool; be sure to test it a few times before you leave. If you think the work is publishable, be sure to talk with your mentor in advance and discuss the work that needs to be done before you leave. Once you leave the company, it's hard to run the program remotely or access the data.

Finishing the internship does not mean the end. In addition to working on a manuscript, you may need to work with your colleagues. By this point, your professional network should be established. Other than being connected on LinkedIn, you should contact your mentor or colleagues occasionally and report your research progress and career plan. If you are interested in working in industry, definitely let them know that you are looking for opportunities. Some interns may land a job after the internship or benefit from the internship during their job hunting. More importantly, you should extend this network to the new students in your group or your school and let the network continue to grow.

**About the Author**

**Bin Chen** is a postdoctoral scholar at Stanford University. During his graduate school at Indiana University, Bloomington, he completed five internships (including one as a contractor) at Novartis, Pfizer, and Merck.
